# Post-endoscopic retrograde cholangiopancreatography pancreatitis

**DOI:** 10.1093/gastro/gou083

**Published:** 2014-11-17

**Authors:** Adarsh M. Thaker, Jeffrey D. Mosko, Tyler M. Berzin

**Affiliations:** Center for Advanced Endoscopy, Division of Gastroenterology, Beth Israel Deaconess Medical Center, Harvard Medical School, Boston, MA, USA

**Keywords:** endoscopic retrograde cholangiopancreatography, pancreatitis, prevention, complications

## Abstract

Acute pancreatitis remains the most common complication of endoscopic retrograde cholangiopancreatography (ERCP). It is reported to occur in 2–10% of unselected patient samples and up to 40% of high-risk patients. The purpose of this article is to review the evidence behind the known risk factors for post-ERCP pancreatitis, as well as the technical and medical approaches developed to prevent it. There have been many advances in identifying the causes of this condition. Based on this knowledge, a variety of preventive strategies have been developed and studied. The approach to prevention begins with careful patient selection and performing ERCP for specific indications, while considering alternative diagnostic modalities when appropriate. Patients should also be classified by high-risk factors such as young age, female sex, suspected sphincter of Oddi dysfunction, a history of post-ERCP pancreatitis, and normal serum bilirubin, all of which have been identified in numerous research studies. The pathways of injury that are believed to cause post-ERCP pancreatitis eventually lead to the common endpoint of inflammation, and these individual steps can be targeted for preventive therapies through procedural techniques and medical management. This includes the use of a guide wire for cannulation, minimizing the number of cannulation attempts, avoiding contrast injections or trauma to the pancreatic duct, and placement of a temporary pancreatic duct stent in high-risk patients. Administration of rectal non-steroidal anti-inflammatory agents (NSAIDs) in high-risk patients is the proven pharmacological measure for prevention of post-ERCP pancreatitis. The evidence for or against numerous other attempted therapies is still unclear, and ongoing investigation is required.

## Introduction

Endoscopic retrograde cholangiopancreatography (ERCP) plays an ever-expanding role in the management of diseases involving the bile duct and pancreatic duct but, as an invasive procedure, it carries significant risks to the patient. The most common complication is acute pancreatitis, which is reported to occur in 2–10% of patients overall (ranging from 2–4% in low-risk patients up to 8–40% in high-risk patients) [1, 2]. A recent meta-analysis of 108 randomized, controlled trials (RCTs) reported an overall incidence of 9.7%, with a mortality rate of 0.7% [3]. This accounts for significant morbidity, occasional mortality, and estimated costs of exceeding US$150 million in the United States each year [3, 4].

Numerous attempts have been made, over several decades, to prevent post-ERCP pancreatitis or limit its severity, although only a few strategies have been proven effective and subsequently accepted into clinical practice. There are several approaches that are employed to reduce the occurrence of this complication. The first is careful patient selection in order to avoid unnecessary exposure to ERCP and its accompanying risks, using instead newer, less-invasive diagnostic modalities when indicated. Second is the use of epidemiological data to identify the most important risk factors for the development of pancreatitis. High-risk patients may warrant specific preventive endoscopic procedures, such as pancreatic duct stent placement. Risk stratification may also prompt referral of high-risk patients to expert providers. Finally, there are ongoing efforts to identify pharmacological agents that provide effective medical prophylaxis against pancreatitis, with promising recent developments regarding non-steroidal anti-inflammatory agents (NSAIDs). The intention of this review article is to describe the mechanisms and risk factors for post-ERCP pancreatitis and to summarize the many efforts being made to prevent this common complication.

## Definition and classification of post-ERCP pancreatitis

Post-ERCP pancreatitis is diagnosed when patients develop signs and symptoms of acute pancreatitis (i.e. abdominal pain) in addition to elevation of pancreatic enzymes. But it is important to consider other causes of post-procedural abdominal pain, such as air insufflation and, less commonly, perforation. Serum amylase levels may be elevated after ERCP in up to 75% of patients, regardless of symptoms [1]. As such, consensus criteria were developed to help standardize the definition and classification of post-ERCP pancreatitis (Table 1). The 2014 revised European Society of Gastrointestinal Endoscopy (ESGE) guidelines recommend that either of two definitions may be used [5]. Neither set of criteria is ideal since, on prospective study, the clinical correlation between them seems to be poor [6].

**Table 1.** Definitions and classifications for post-ERCP and acute pancreatitis

	Mild	Moderate	Severe
Cotton criteria	a) New or worsened abdominal pain and b) amylase >3 times normal limit 24 hours after the procedure and c) requiring hospital stay or extension of stay by 2–3 days	Requiring 4–10 day hospitalization	a) >10 day hospitalization or b) development of a complication (e.g. necrosis or pseudocyst) or c) need for intervention (drainage or surgery)
Revised Atlanta Classification^a^	Two out of three: a) pain consistent with acute pancreatitis b) amylase or lipase >3 times normal limit c) characteristic findings on abdominal imaging and d) no organ dysfunction or complications	a) Transient organ failure <48 hours or b) local or systemic complications without persistent organ failure	a) Persistent single or multi-organ failure >48 hours or b) present or persistent systemic inflammatory response syndrome (SIRS)

^a^These criteria were developed for acute pancreatitis, not specifically for post-ERCP pancreatitis.

In the criteria developed by Cotton *et al*. in 1991, mild post-ERCP pancreatitis was defined as abdominal pain suggestive of pancreatitis requiring new hospitalization or extension of hospital stay for 2–3 days and a serum amylase at least three times the upper limit of normal, 24 hours after the procedure [7]. Modifications to this definition have been proposed to allow lipase as an alternative to amylase and defining clinical pancreatitis specifically as “new or worsened abdominal pain” to account for patients who undergo ERCP for pre-existing pain from acute and/or chronic pancreatitis [8]. Moderate severity is defined by the need to stay in hospital for between 4 and 10 days. Severe post-ERCP pancreatitis is defined as the need for a hospital stay longer than 10 days, or by the development of a complication such as necrosis or pseudocyst, or need for intervention (drainage or surgery) [2, 7].

The second definition is in the 2012 revised Atlanta Classification of acute pancreatitis, an international consensus statement, in which the diagnosis of acute pancreatitis requires two of three features: (i) abdominal pain consistent with acute pancreatitis, (ii) serum lipase or amylase greater than three times the upper limit of normal and (iii) characteristic findings of acute pancreatitis on contrast-enhanced computerized tomography (CT) scan, magnetic resonance imaging (MRI), or transabdominal ultrasound [9]. Mildly severe acute pancreatitis is characterized by absence of accompanying organ failure, local complications, or systemic complications. Moderate acute pancreatitis includes transient organ failure (<48 hours) or local or systemic complications without persistent organ failure (e.g. fever, leukocytosis, exacerbation of chronic lung disease). Severe acute pancreatitis is characterized by persistent organ failure (>48 hours) or the presence of a systemic inflammatory response syndrome (SIRS) at any time, given the high risk of progression to persistent organ failure [9]. This definition is limited, in that it was not developed primarily for post-ERCP pancreatitis, but for all-cause pancreatitis. Most of the studies described here used the Cotton- or similar criteria to define and classify post-ERCP pancreatitis, but the variables in the revised Atlanta Classification are increasingly used in research studies.

## Pathogenesis of post-ERCP pancreatitis

The mechanisms that cause pancreatitis are poorly understood, but numerous theories have been proposed. The common endpoint is the activation of inflammatory pathways. Mechanical obstruction of the papilla or pancreatic sphincter by instrumentation, hydrostatic injury from the injection of contrast, water, and chemical or allergic injury from contrast injection are possible mechanisms that may occur during ERCP. Injury, edema, or perforation of the pancreatic sphincter, bile duct, or ampulla by instrumentation or from thermal injury produced by electrocautery, may also obstruct the flow of pancreatic secretions. These same mechanisms may produce intraluminal activation of proteolytic enzymes and resultant injury. It has also been suggested that infection plays a part, due to the possible introduction of luminal contamination into the ducts [1, 10].

The resultant cascade of inflammation includes the premature intra-acinar activation of zymogens into proteolytic enzymes, chemo-attraction of inflammatory cells, and the release of inflammatory mediators and cytokines. This cascade can be limited to local inflammation or initiate a systemic inflammatory response syndrome (SIRS) [1, 11].

## Risk factors and predictors for post-ERCP pancreatitis

Numerous prospective single-center and multi-center studies and meta-analyses have been carried out to identify risk factors for post-ERCP pancreatitis. These can be classified into patient-related factors, provider-related factors, and risks related to the procedure itself (Table 2).

**Table 2.** Risk factors for post-ERCP pancreatitis

Patient-related	Provider-related	Procedure-related
Young age	Experience (maybe)	Difficult cannulation
Female		Number of cannulation attempts
Suspected SOD dysfunction		Pancreatic duct cannulation
Prior post-ERCP pancreatitis		Pancreatic duct injection
Normal serum bilirubin		Pre-cut sphincterotomy
Previous recurrent pancreatitis		Ampullectomy

SOD = Sphincter of Oddi

### Patient-related risk factors

Several factors that place the patient at increased risk of post-ERCP pancreatitis have been identified in descriptive studies. In most of the recent prospective studies or in meta-analyses, characteristics that were statistically significant by multivariate analysis included younger age, female gender, suspected or proven sphincter of Oddi dysfunction, prior post-ERCP pancreatitis, a normal serum bilirubin, and recurrent pancreatitis [1, 2, 12–15]. The use of certain pancreato-toxic drugs (i.e. valproic acid, azathioprine or estrogen), smoking, and the absence of a common bile duct stone have also been implicated as potential risk factors in retrospective studies but require further investigation.

Importantly, risk factors for post-ERCP pancreatitis have been shown to be additive and perhaps synergistic. Taken together, the above variables profile a patient who may be especially prone to inflammation of the pancreas after an insult, and therefore warrants special caution; for example, one study demonstrated that a young female patient with suspected sphincter of Oddi dysfunction and normal serum bilirubin was shown to have a risk of pancreatitis of 18%, compared with 1.1% in an otherwise healthy patient. If this same patient had no evidence of bile duct stones, and if cannulation was difficult during the procedure, the risk of pancreatitis increased to over 40% [2, 12, 16]. It is unclear why patients with these characteristics—some of whom may not have a definitive indication for ERCP to begin with—are paradoxically at the highest risk for a complication, but the importance of identifying the risk factors is clear.

The wide range of reported incidence of post-ERCP pancreatitis over risk groups in observational studies prompted a 2014 systematic review including 108 RCTs that covered 13 296 patients [3]. The overall incidence of post-ERCP pancreatitis was 9.7%, of which 8.6% of cases were mildly severe, 3.9% were moderate, and 0.8% were severe. The incidence of all-severity post-ERCP pancreatitis in high-risk patients was 14.7%. This study also found, however, that the incidence of severe post-ERCP pancreatitis (0.5% of all ERCPs performed) did not differ between patients in a high-risk subgroup and non-risk-stratified RCTs (0.8% *vs.* 0.4%, respectively), perhaps due to heterogeneity between the RCTs regarding the risk assessment of patients.

### Provider-related risk factors

There is conflicting evidence regarding the influence of endoscopist experience, the procedure volume of a specific center, and the involvement of trainees on the risk of post-ERCP pancreatitis [2]. In Italy in 2007, a large prospective study comparing the experience of endoscopy centers and providers found no difference, between high- and low volume centers, in the incidence of post-ERCP pancreatitis after 3635 procedures. This study also did not find a statistically significant difference in the rates of post-ERCP pancreatitis between expert and non-expert endoscopists [17]. Another large, prospective, multicenter trial also found that the number of ERCPs performed by an endoscopist did not affect the rate of perceived pancreatitis after biliary sphincterotomy, although endoscopists who performed fewer than one sphincterotomy per week identified a significantly higher rate of overall complications [8].

### Procedure-related risk factors

Several technical factors are known to increase the risk of post-procedure pancreatitis in multivariate prospective studies or meta-analyses. Difficult cannulation (characterized by a greater number of attempts or longer time needed to successfully cannulate the bile duct) can result in trauma to the ampulla and increases the risk of subsequent pancreatitis independent of other factors [1, 8, 13]. The risk increases with a greater number of cannulation attempts, with one study which included all types of intra-ERCP procedures describing a 3.3% pancreatitis rate in patients requiring less than five attempts at cannulation, 9% for 6–20 required attempts, and 14.9% if more than 20 attempts were necessary [1]. Spending more than 10 minutes attempting cannulation also increases the risk, although a recent prospective study demonstrated that even a duration exceeding 5 minutes may increase the risk of post-ERCP pancreatitis when compared with shorter-duration attempts [18]. Pancreatic duct cannulation, more than one passage of a pancreatic guide wire, pancreatic duct injection/pancreatogram, pre-cut sphincterotomy (a last-resort technique to gain access to the bile duct after other cannulation methods have failed), pancreatic sphincterotomy, and ampullectomy have also repeatedly been identified as independent risk factors for post-ERCP pancreatitis [1, 5, 14, 15, 17, 19–21]. High-risk procedural factors should warrant consideration of additional measures to limit the occurrence or severity of post-ERCP pancreatitis, as described below.

### Predictors of post-ERCP pancreatitis

Observational studies have determined that serum amylase or lipase levels of less than 1.5 and 4 times the upper limit of normal, respectively, 2–4 hours post-ERCP, have a very high negative predictive value for post-ERCP pancreatitis. Guidelines have therefore suggested that, if a patient is to be discharged the day of the procedure but is experiencing pain, serum amylase or lipase levels, tested at 2–6 hours post-ERCP and found to be below these cut-offs, is reassuring enough to allow safe discharge home [5].

## Preventive strategies

A variety of strategies have been investigated for reducing the risk of post-ERCP pancreatitis. The best-supported approach involves careful patient selection, use of certain procedure-related techniques, and pharmacological agents, for which the evidence for use of rectal NSAIDs is the most widely accepted (Table 3).

**Table 3.** Recommended strategies for prevention of post-ERCP pancreatitis

All patients	In high-risk patients	Unclear efficacy
Careful patient selection	Pancreatic duct stent placement	Nitrates
Consider alternative modalities	Single-dose rectal indomethacin	Protease inhibitors
Provider training and experience		Intravenous fluid hydration
Guide wire-assisted cannulation		

### Patient selection

Given the morbidity and cost associated with ERCP, careful selection of patients is paramount. To reflect this, the latest consensus statements and guidelines state that ERCP should be reserved for specific indications and used primarily as a therapeutic procedure as part of a risk-stratified, evidence-based approach to suspected pancreaticobiliary diseases [22, 23]. The diagnostic role of ERCP has been largely supplanted by safer alternative technologies, such as endoscopic ultrasound (EUS) and magnetic resonance cholangiopancreatography (MRCP) [22, 24, 25].

For the evaluation of suspected choledocholithiasis, MRCP was reported in systematic reviews to have a sensitivity of 85–92% and a specificity of 93–97% for choledocholithiasis [26, 27]. EUS has a reported sensitivity of up to 97% and a negative predictive value approaching 100%, while avoiding the need to instrument the common bile duct. EUS is also extremely sensitive for stones smaller than 5mm [24, 28]. Helical CT scanning has a reported sensitivity of 65–88% and a specificity of 73–97% for bile duct stones, although this is not a first-line diagnostic tool for biliary pathology due to the risks from radiation exposure and the lower sensitivity for detecting bile duct stones [28].

Several epidemiological studies have reported a steady fall in the number of diagnostic-ERCP procedures being performed, even though the total number of ERCPs performed is rising [24, 25, 29]. This is probably due to the wider availability and acceptance of these alternative diagnostic technologies for pancreaticobiliary disease.

### Procedure-related strategies

Endoscopic technique and choice of instrument play important roles in the risk of post-ERCP pancreatitis. Wire-guided cannulation of the biliary duct and prophylactic pancreatic stent placement in high-risk patients are the two techniques which have been studied most extensively.

#### Guide wire-assisted cannulation

**Figure 1.**
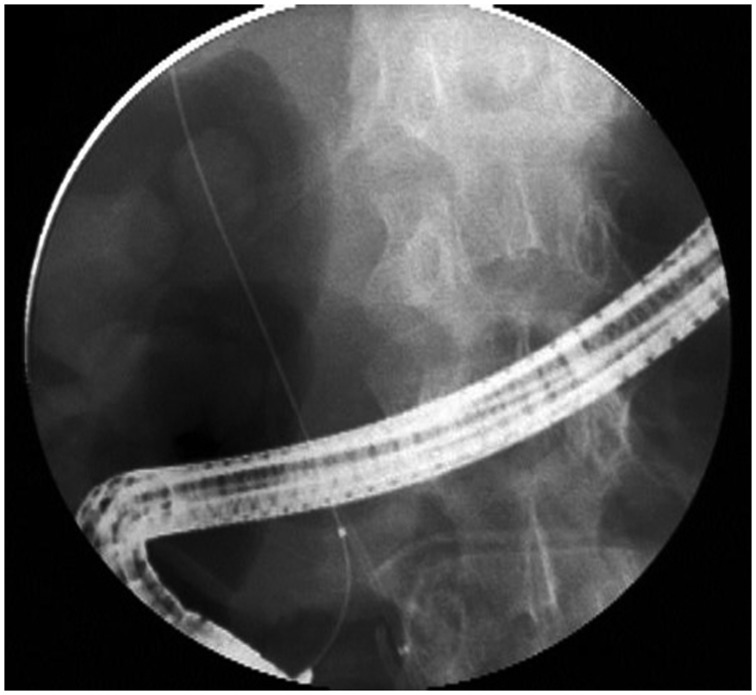
Fluoroscopic image of wire-guided biliary duct cannulation.

Guide wire cannulation refers to the insertion of a hydrophilic tipped guide wire selectively into the bile duct or pancreatic duct and obtaining fluoroscopic confirmation of the wire placement before inserting a sphincterotome or catheter over the guide wire through the ampulla (Figure 1). The use of a guide wire to cannulate the bile duct is an alternative to contrast-assisted cannulation. The theory behind the use of a guide wire is that it may improve biliary cannulation success, reduce papillary trauma, and avoid inadvertent contrast injection into the pancreatic duct, thereby reducing the risk of post-ERCP pancreatitis. The guide wire is smaller than the larger catheter or sphincterotome and therefore may pass more easily through the ampulla. Potential risks of guide wire-assisted cannulation include creating a false passage/tract, intramural dissection, perforation, and pancreatic ductal injury [19]; however, these complications are rare and can be limited by using a soft-tipped wire with a hydrophilic coating.

While the rate of successful biliary cannulation was shown to be higher with the use of a guide wire, there is mixed evidence regarding the efficacy of this cannulation method in reducing post-ERCP pancreatitis [21, 30–32]. A meta-analysis published in 2013, which included 12 randomized, controlled trials and 3450 patients, reported a significant decrease in the rates of post-ERCP pancreatitis through the use of guide wire-assisted cannulation, compared with conventional contrast-assisted cannulation (3.5% *vs.* 6.7%, respectively) [30]. The guide wire-assisted cannulation technique was also associated with greater primary cannulation success and fewer pre-cut sphincterotomies, both factors that may reduce the risk of post-ERCP pancreatitis. Wire-guided cannulation also reduced the rate of post-ERCP pancreatitis in cases with inadvertent manipulation of the pancreatic duct (i.e. by accidental wire insertion or contrast injection); however, the reduction in post-ERCP pancreatitis was only seen in the five ‘non-crossover' studies, which did not allow for other endoscopic risk reduction techniques [30]. Studies that permitted prophylactic pancreatic duct stenting found no statistically significant difference in the rates of post-ERCP pancreatitis between the guide wire-assisted and contrast-assisted techniques (4.8% *vs.* 5.5%).

Another recently published prospective study of 1249 patients also found no significant difference between wire-guided *vs.* contrast-assisted techniques in the development of pancreatitis [31]. Nonetheless, given its demonstrated improvement in cannulation success and the evidence that it may reduce the risk of post-ERCP pancreatitis without significant harm, guide-wire assistance is generally recommended for deep biliary cannulation [5].

A pancreatic guide wire (double-guide wire technique) may also be used to facilitate deep biliary cannulation by straightening the papillary anatomy and reducing inadvertent cannulation of the pancreatic duct; however, this technique increases the risk of post-ERCP pancreatitis, so its use should be restricted, and prophylactic pancreatic duct stenting, described below, should be strongly considered [5].

#### Prophylactic pancreatic duct stenting

**Figure 2.**
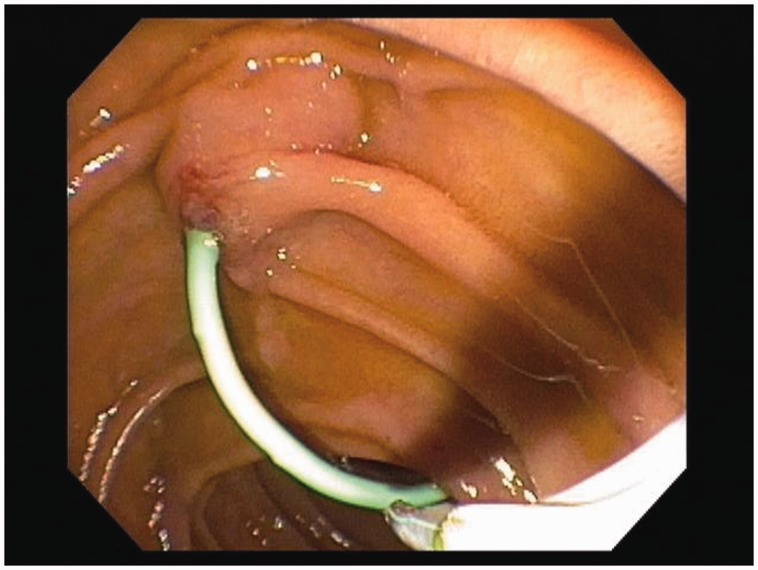
Pancreatic duct stent in place, seen emerging into the duodenum.

Pancreatic duct (PD) stenting has been shown to significantly reduce the rates of post-ERCP pancreatitis. A stent placed across the ampulla and pancreatic sphincter into the pancreatic duct is presumed to maintain the flow of pancreatic secretions across any flow disruptions caused by injury or edema of these structures (Figure 2).

There has been substantial evidence, over the past few decades, to support the efficacy of stent placement for prophylaxis against post-ERCP pancreatitis, especially in high-risk patients. A recent meta-analysis, reviewing 14 randomized controlled trials with a total of 1541 patients, demonstrated a statistically significant reduction in post-ERCP pancreatitis for both high-risk patients and mixed-risk groups and an overall relative risk reduction of ∼ 63% [33]. This study confirmed the findings and recommendations of previous meta-analyses, while also demonstrating that PD stenting reduced the occurrence of mild, moderate, and severe post-ERCP pancreatitis [21, 33]. Despite substantial evidence of its benefit, there are several areas of uncertainty related to pancreatic duct stenting; specifically, there is no clear consensus on how to decide which patients should receive a stent or what type and size of stent to use. While the ideal choice of stent is not definitively known, short, 5-Fr diameter, plastic pancreatic stents seem to be safe, the most efficacious, and easiest to use [5]. PD stent placement may also be more challenging in patients with small or tortuous ducts, with a reported overall failure rate of 5–10%, even at advanced centers [1]. There is the need for—and cost of—a follow-up procedure to remove the stent, generally recommended within 1–2 weeks to avoid the potential complication of stent occlusion or inward stent migration [34]. The success of stent insertion is operator-dependent and some endoscopists may be less comfortable with this technique. The risk of pancreatitis after failed pancreatic stenting attempts is high, and the studies that reported the benefits of PD stenting were mostly performed by advanced centers with high patient numbers and expert endoscopists [35]. Therefore, the benefits of a PD stent may greatest when the procedure is performed successfully by an experienced provider; for this reason, proper training, experience, and technique are very likely to be important in achieving the intended risk reduction with PD stent placement.

A recently published article suggested a potential role for ‘salvage' ERCP with PD stent placement, to help reduce the duration and severity of pancreatitis in patients recognized to have post-ERCP pancreatitis [36]. Salvage ERCP was considered in two groups of patients: (i) patients who did not have a PD stent placed in the original procedure and (ii) patients who had a PD stent placed, which subsequently migrated. There is not yet sufficient evidence to recommend widespread use of salvage ERCP for PD stent placement.

#### Other techniques

Pre-cut sphincterotomy refers to several ‘last resort' techniques to gain deep biliary access for difficult cannulations. Its use is associated with an increased risk of post-ERCP pancreatitis, although this may be attributable to the failed cannulation attempts that often precede a pre-cut sphincterotomy, rather than the procedure itself. There is a variety of techniques used to perform a pre-cut sphincterotomy, but there is some evidence that needle-knife fistulotomy is associated with the lowest risk of post-ERCP pancreatitis, and is suggested by the ESGE as the preferred initial technique [5].

Endoscopic papillary balloon dilation (EPBD), or sphincteroplasty, is an alternative to sphincterotomy in assisting with the extraction of large stones. The advantages are preservation of sphincter of Oddi function, reduced bleeding compared with traditional sphincterotomy (preferable for patients with severe coagulopathy), and utility in patients with altered anatomy (e.g. after Billroth II gastric bypass surgery) [5]; however, sphincteroplasty alone seems to have a lower success rate for the removal of biliary stones and a higher incidence of post-ERCP pancreatitis, so this technique is rarely used outside the above situations. There is evidence that performing dilation over a short time period (<1 minute) is associated with an increased risk of post-ERCP pancreatitis so, if used, it is recommended that the time taken for balloon dilation be longer than one minute [5].

### Medical management

Given the challenges faced in using endoscopic techniques to reduce the risk of post-ERCP pancreatitis, considerable efforts have been made to find a pharmacological agent to be given before or after the procedure as prophylaxis. Unfortunately, many of the agents that showed initial promise in animal studies or small randomized trials failed to show a consistent benefit in subsequent large, multicenter studies. The rationale behind many of the pharmacological interventions has been the prevention or interruption of the cascade of pathophysiological events that are hypothesized to cause pancreatitis after ERCP. The agents can therefore be grouped by their postulated mechanisms of action.

#### Reduction of sphincter of Oddi pressure or spasm

Several agents have been studied to theoretically improve pancreatic drainage by improving flow through the sphincter of Oddi to help prevent pancreatitis. This includes nifedipine, botulinum toxin, topical lidocaine (applied to the papilla), secretin, and phosphodiesterase Type 5 inhibitors, none of which has been reliably shown to have a benefit [20]. In this category, only nitrates have showed some promise of efficacy.

Nitrates given by topical, sublingual, transdermal, and intravenous routes have been studied extensively, with several randomized, controlled trials yielding conflicting results. Four meta-analyses showed a significant risk reduction for post-ERCP pancreatitis with nitrate therapy, but a fifth showed no difference [37–40]. The most recent meta-analysis included 11 randomized placebo-controlled studies and found a significant decrease for overall post-ERCP pancreatitis in patients receiving nitrates, but no reduction in moderate- and high-severity pancreatitis [40]; however, only 3 of the 11 studies in this analysis showed a significant difference between the nitrate and placebo, and some of the studies had an unusually high background rate of pancreatitis for average risk patients, thereby limiting conclusions about high-risk patients [1]. Another double-blinded, randomized, controlled trial published in 2014 compared rectal indomethacin plus sublingual nitrate against rectal indomethacin and placebo prior to ERCP for 300 patients at a single center [41]. This study found a significant reduction in the incidence of post-ERCP pancreatitis for a combination of the nitrate with indomethacin than for indomethacin alone. Given the conflicting evidence behind nitrates and the risk of side-effects such as headache and hypotension, large multicenter studies are still required before definitive conclusions can be drawn.

#### Reduction of pancreatic secretions

Studies of agents that inhibit pancreatic secretions—including somatostatin and its synthetic analogue, octreotide—have produced conflicting results concerning their ability to prevent post-ERCP pancreatitis [42–44]. The value of these agents is not yet certain and they are not currently recommended for routine use.

#### Inhibition of proteolytic enzymes

Protease inhibitors, such as gabexate, nafamostat, and ulinastatin, have been investigated in pursuit of the theory that premature activation of proteolytic pancreatic enzymes causes post-ERCP pancreatitis. Some studies have demonstrated a small benefit, while others revealed no effect. A 2011 meta-analysis, including 4966 patients in eighteen studies, found an overall significant but small risk reduction, with a number needed to treat of 34.5 [45]. Subgroup analysis showed no significant efficacy for gabexate and mixed results for ulinastatin. A subsequent trial comparing gabexate, ulinastatin, and placebo showed that gabexate was effective in reducing post-ERCP pancreatitis [46]; however this study was non-randomized, retrospective in nature, and the three study groups underwent their procedures at different times over a ∼ 5.5 year period. Additionally, the study required 24-hour infusions of the agents due to their short half-life, which may not be practical from a cost or resource standpoint. Therefore, the value of these and other agents that may inhibit the activation of pancreatic enzymes is not well established.

#### Inhibition of the inflammatory cascade

Numerous anti-inflammatory agents—including NSAIDs, steroids, anti-metabolites (allopurinol, 5-fluorouracil), anti-oxidants, and other medications with anti-inflammatory properties (e.g. heparin, risperidone)—have been studied for prophylaxis against post-ERCP pancreatitis. The objective of these agents is to disrupt the inflammation cascade that is presumed to be the common final pathway in the development of pancreatitis. Study medications were chosen, based on their effect on inflammatory mediators such as phospholipase A2, leukocytes, other inflammatory cells, and cytokines which were implicated in earlier *in vitro* studies [11]. Out of the many trials, rectal NSAIDs (mainly indomethacin and diclofenac) emerged as the most reliable and beneficial agents for the prevention of post-ERCP pancreatitis.

An important multicenter, randomized, placebo-controlled, double-blinded clinical trial of 602 patients was published in the *New England Journal of Medicine* in 2012 and confirmed preliminary evidence suggesting a benefit for post-procedure rectal indomethacin [4]. This study selectively enrolled patients determined to be at high risk, based on factors including any one or more of the following major criteria: (i) suspected sphincter of Oddi dysfunction, (ii) a history of post-ERCP pancreatitis, (iii) pancreatic sphincterotomy, (iv) pre-cut sphincterotomy, (v) more than eight cannulation attempts, (vi) pneumatic dilation of an intact biliary sphincter or (vii) ampullectomy. Patients were also included if they met two or more of the following minor criteria: (i) females younger than 50 years age, (ii) history of two or more recurrent episodes of pancreatitis, (iii) three or more injections of contrast agent to the tail of the pancreas, (iv) excessive contrast injection to the pancreatic duct detected by acinar opacification or (v) if cytology was obtained from the pancreatic duct using a brush (thereby inducing trauma). The rate of post-ERCP pancreatitis was 9.2% in the group receiving a single dose of post-procedure rectal indomethacin, against 16.9% in the placebo group, a difference which was statistically significant. Prophylactic indomethacin also decreased the severity of pancreatitis, and the benefit of indomethacin was similar, whether or not the patients received pancreatic duct stenting.

Subsequently, two meta-analyses published in 2014, which included nine randomized controlled trials and 2133 patients, determined that a single rectal dose of either indomethacin or diclofenac immediately before or after ERCP was equivalent in efficacy [47, 48]. The overall incidence of post-ERCP pancreatitis was significantly decreased, with reported risk ratios of 0.44 and 0.51, and numbers needed to treat of 11 and 14, respectively, in the two studies. No complications related to NSAID use were reported, and the NSAIDs reduced the risk in mixed-risk as well as in high-risk groups.

A significant benefit of rectal NSAIDs, compared with the other studied agents, is that they are effective even when administered after the procedure, so that the provider can respond to procedure-related risk factors (such as difficult cannulation, pancreatic duct injection, etc.) that were not predictable before the ERCP. NSAIDs are also inexpensive, easy to administer, and have no significant adverse effects with a single dose [4]. Compared with other well-validated preventive measures, such as pancreatic duct stenting, rectal NSAIDs do not rely on operator technique or expertise, and therefore currently represent the best-established preventive option for high-risk patients. Controlled studies comparing rectal NSAIDs or pancreatic duct stenting alone, against a combination of the two, are not yet available.

While the European Guidelines recommend routine use of rectal NSAIDs in ERCP, there is not yet a consensus in the United States regarding their universal or selective use in this situation [5].

#### Aggressive hydration

Intravenous fluid resuscitation remains the principle evidence-based treatment of acute pancreatitis from any cause. It is thought to counteract microvascular hypoperfusion and the resulting cell injury [49]. The use of lactated Ringer’s solution (as opposed to normal saline) may further avoid acidosis, which seems to activate pancreatic zymogens into proteolytic enzymes that damage the pancreas and cause inflammation and pancreatitis [50]. A pilot study published in 2014 found that aggressive hydration with lactated Ringer’s solution appeared to reduce the post-ERCP pancreatitis rates in inpatients, without significant complications from volume overload [49]. Additional studies are needed to further evaluate the role of peri-procedural hydration which, like NSAIDs, could represent another inexpensive and relatively simple prophylactic intervention.

#### Other agents

Only one study concluded that ceftazidime decreased the risk of pancreatitis and there are no reliable confirmatory studies to support the efficacy of antibiotics [20, 51]. Additionally, use of high-osmolality *vs.* low-osmolality contrast does not seem to affect the risk of post-ERCP pancreatitis [52]. Various other agents have been studied and are probably ineffective in preventing post-ERCP pancreatitis.

## Our clinical practice

At our institution—a high-volume tertiary referral center for pancreaticobiliary disease—our practice with respect to the prevention of post-ERCP pancreatitis continues to progress as new evidence becomes available. Before each procedure, all patients undergo a thorough assessment for possible risk factors for post-ERCP pancreatitis. Sphincterotomes are used exclusively for cannulation in patients without prior sphincterotomies. Both wire-guided and contrast-injection techniques for cannulation are used, at the discretion of our endoscopists. Rectal indomethacin (post-procedure) and intravenous normal saline or lactated Ringer's solution (pre- and post-procedure) are given to patients who are deemed to be at high risk (i.e. young, female, possible sphincter of Oddi dysfunction, previous episode of post-ERCP pancreatitis and/or a normal bilirubin) and to patients undergoing high-risk procedures (e.g. pancreatic sphincterotomy, sphincter of Oddi manometry and/or ampullectomy). In our practice, pancreatic duct stenting is generally used in cases of difficult cannulation, to assist with biliary access, or in cases focused on pancreatic duct interventions, such as stone removal. In these cases, the stent is generally left in place for 1–2 weeks if the patient or procedure had high-risk features for pancreatitis. Finally, during our ERCPs, pancreatic duct injections are minimized, and pre-cut sphincterotomies are reserved for emergency biliary access after all other methods have failed.

## Conclusion

Despite advances in knowledge behind the mechanisms and risk factors for post-ERCP pancreatitis, the incidence of this condition is still high and is the most common complication of ERCP. The primary approach to prevention is through careful patient selection, sound endoscopic technique, and evidence-based medical management. Ongoing identification and special attention to risk factors for post-ERCP pancreatitis is vital, in order to optimize patient selection and to guide specific procedure techniques and other prophylactic measures. There are several mechanisms that contribute to the development of pancreatitis and that can be targeted for protective endoscopic or medical therapies. Preventive measures include procedural techniques such as the use of a guide wire cannulation, minimizing the total number of cannulation attempts, and avoiding contrast injections or trauma to the pancreatic duct. The placement of temporary pancreatic stents and administration of rectal NSAIDs in high-risk patients remain the interventions with proven efficacy and thus should be incorporated into clinical practice. High-quality studies are still needed to better evaluate other medical therapies.

*Conflict of interest statement.* none declared.
